# Percutaneous CT-Guided Biopsy of the Craniovertebral Junction: Safety, Diagnostic Yield, and Technical Notes

**DOI:** 10.3390/diagnostics12010168

**Published:** 2022-01-11

**Authors:** Paolo Spinnato, Eugenio Rimondi, Giancarlo Facchini

**Affiliations:** Diagnostic and Interventional Radiology, IRCCS Istituto Ortopedico Rizzoli, 40136 Bologna, Italy; eugenio.rimondi@libero.it (E.R.); giancarlo.facchini@ior.it (G.F.)

**Keywords:** bone neoplasms, image-guided biopsy, atlanto-axial joint, atlanto-occipital joint

## Abstract

The craniovertebral junction defined as the occiput, the atlas, and the axis is a complex bony region that contains vital neural and vascular structures. We report the experience of a single academic institution regarding CT-guided biopsy of this skeletal region. We reviewed all of the CT-guided biopsies performed in our department, completed in the craniovertebral junction. We collected data in regard to biopsy procedures, patients’ vital statistics, and histopathological diagnosis. In total, 16 patients (8M and 8F; mean age 52; range 16–86 years old) were included in this series. In eight patients, the lesions were located in the atlas vertebra (8/16—50%), in six patients in the axis (37.5%), and in two patients in the occiput (12.5%). No complications were observed during or after the procedures. All of the procedures were technically successful. The biopsy was diagnostic in 13/16 patients (81.3%): four metastatic lesions (25%—three breast and one prostate cancers), four multiple myeloma bone lesions (25%), three aneurismal bone cysts (18.8%), one aggressive hemangioma (6.3%), and one pseudogout (6.3%). Moreover, in two-thirds (66.6%) of non-diagnostic histological reports, malignancies were excluded. CT-guided percutaneous biopsy is a safe tool and allows obtaining a histological diagnosis, in most cases, even in the most delicate site of the human skeleton—the craniovertebral junction.

## 1. Introduction

Percutaneous biopsy is routinely performed in the axial and appendicular skeleton as the first diagnostic approach for histopathological assessment of bone lesions, mainly neoplasms and infections [1]. 

Computed tomography (CT)-guided biopsy is proved to be an effective and safe tool for the diagnosis of spinal bone lesions [2,3,4,5]. Moreover, CT-guided biopsy showed a higher success rate than fluoroscopic-guided biopsy of the spine, and it is by far less invasive than open surgical biopsy.

Despite the proven effectiveness and safety of this diagnostic procedure, there is a lack of data in the literature in regard to its application in the most delicate site of the human skeleton—the craniovertebral junction (CVJ).

CVJ is a complex bony structure defined as the occiput (or C0), the atlas (C1), and the axis (C2). The occiput has two condyles articulated with the atlas. The atlas has no body and is composed of posterior and anterior arches and two lateral masses. The axis has a body, a posterior arch, and an odontoid process (the “dens”). This anatomical region contains two major joints: the atlanto-occipital joint and the atlanto-axial joint. A minor joint is also present between the odontoid process of the axis and the anterior arch of the atlas. These structures are stabilized by numerous muscular and ligamentous components. CVJ connects the upper cervical spine to the skull, and therefore the spinal canal to the foramen magnum.

CVJ contains vital neural structures (vertebral arteries, brain stem, spinal cord) while achieving the most mobility of any spinal segment [6]. Indeed, most functions such as cranial extension, flexion, and axial rotation are accomplished in the CVJ area [6,7].

The combination of bony, muscular, and neurovascular vital structures crowded in a deep and narrow space renders surgical and percutaneous approaches to the CVJ area hard and risky.

In a large series focused on CT-guided biopsy of the spine, among 430 patients, procedures were performed within the atlas or axis in three cases only (3/430—0.7%) [8]. Another study focused on CT-guided biopsy of the cervical spine, reported nine biopsies completed within the atlas or axis (9/73—12.3%) [9]. In the only series of CT-guided biopsies of the skull, lesions were located in the occiput only in three cases (3/14—21.4%) [10].

Our study aims to assess the feasibility, safety, and diagnostic yield of CT-guided biopsy performed within CVJ.

## 2. Materials and Methods

We retrospectively reviewed all of the CT-guided biopsies performed in our institution completed within CVJ (C0-C3) from the year 2006. We collected vital statistics of each patient. We reviewed all of the histopathological reports obtained from biopsy specimens, to assess the diagnostic yield. Moreover, clinical reports and imaging studies available for each patient have been reviewed as well.

Two expert musculoskeletal radiologists, respectively, with 10 and 14 years of experience reviewed all imaging studies and CT-guided biopsy image scans on a PACS (Carestream Vue PACS v. 11.4.1.1102). 

### 2.1. Procedure Techniques

Procedures were performed by expert musculoskeletal interventional radiologists (with experience of at least 5 years and 600 biopsy procedures performed). All of the biopsy requests were carefully evaluated by radiologists and approved only if a safe needle approach to the lesion was feasible, avoiding vital structures damaging. The needle approach was chosen according to spinal surgeons′ indications for a potential future surgical procedure (in case of suspected primary bone neoplasms) and according to the safest approach concerning vital structures. Patient position on the CT table (supine/prone) depended on the chosen approach.

All biopsies were carried out with a 12Gauge or 14Gauge needle, Bonopty Insertion Set (Apriomed, Uppsala, Sweden). We used the small-caliber set (14G) only when the site, size, or approach did not allow the use of a larger needle.

CT scans were acquired with Brilliance CT 16 slice (Philips Medical Systems, Cleveland, OH, USA) equipment.

Radiologists accurately reviewed all images, especially CT and MRI, to assess the site of vital neural structures and to plan the procedure.

A scout-view radiogram was taken, focusing on the affected area. The setting of the thickness and slice interval was set with 1 mm thickness and 1 mm interval. An angulated approach was chosen by tilting the CT gantry parallel to the upper and lower surface of the target lesion if needed. If necessary, the pre-procedural CT scan was conducted with contrast media injection, to depict the exact position of vascular structures and their proximity to the target lesion. 

Under CT guidance, the best insertion point was selected and marked on the skin. Then, needle insertion, with coaxial or tandem technique, was performed under sequential scans. A low dose scan was used to monitor the needle approach and progression to the target lesion, to reduce the dose absorbed by the patient. At least two samples were collected. Samples were sent to the pathologist in saline solution. A swab sample was obtained for microbiology to assess the presence of infections.

All biopsies were carried out after informed consent was obtained.

The type of anesthesia carried out as well as the total duration time of procedures were recorded.

The approach to the target lesion was recorded and schematized as follows: posterior, anterior/transoral, and lateral.

At the end of the procedure a final CT scan, after the removal of the needle, was obtained to check for immediate complications such as local hematoma.

### 2.2. Outcome Assessment

CT-guided biopsy complications were documented according to the Society of Interventional Radiology classification [11]. Patients were clinically monitored in the spinal unit of our institution, for a minimum of 12 h after each procedure, to observe any evidence of acute complications (e.g., hematoma formation, neurologic injuries). Patients were clinically evaluated by spinal surgeons before discharge. The electronic medical records were reviewed for evidence of delayed complications within 30 days of the biopsy. 

Biopsies were considered technically successful if the needle was visualized within the target lesions on CT-scan images, and core specimens were obtained. 

CT-guided procedures were considered diagnostically successful if the histopathological analyses on biopsies specimens allowed a final diagnosis to be obtained.

## 3. Results

In total, 16 patients (8M and 8F—mean age 52, range 16–86 years old) were included in the study. In eight patients, the lesions were located in the atlas (8/16—50%), in six patients in the axis (37.5%), and in two patients in the occiput (12.5%). One of the lesions located in the occiput (1/2—50%) involved also the atlas. Moreover, two of the lesions located in the atlas (2/8—25%) involved also the axis.

### 3.1. CT-Guided Biopsy Outcomes

No complications were observed during or after the procedures, at both early and late control within 30 days of the biopsy.

The CT-guided biopsies were technically successful in all cases (16/16—100%), with the needle placed inside the target lesions as documented by CT scans.

CT-guided biopsies were diagnostically successful in 13/16 patients (81.3%). Indeed, histopathological diagnoses were obtained on biopsy specimens of 13 patients. The histological diagnoses obtained included four metastatic lesions (4/16—25%—three breast and one prostate cancers), four multiple myeloma bone lesions (25%), three aneurismal bone cysts (18.8%), one aggressive hemangioma (6.3%), and one pseudogout (6.3%). 

Moreover, in two-thirds (66.6%) of non-diagnostic histological reports, malignancies were excluded, even if the exact histopathological diagnosis was not reached. The two patients were evaluated with MRI follow-up controls and clinical visits, and follow-up time of 3 and 1 years, respectively, from biopsy. We did not have further clinical or radiological data regarding the other patient with biopsy non-diagnostic (1/3—33.3%).

### 3.2. CT-Guided Technical Data

**Figure 1:**
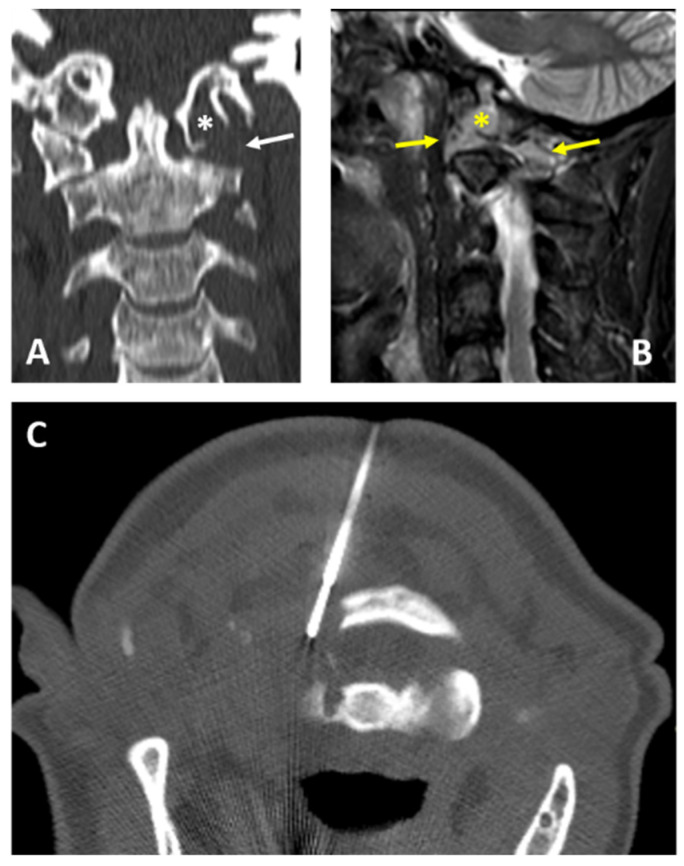
A 52-year-old woman: CT frontal view (**A**); MRI T2w fat sat sagittal view (**B**) show osteolytic lesion involving the left condyle of the occiput (white and yellow asterisks) and both the anterior and posterior arch of the atlas (white and yellow arrows). CT-guided biopsy (**C**) was performed with a posterior approach using a 14G needle (Bonopty). The final histopathological diagnosis on the biopsy specimen was multiple myeloma.

**Figure 2:**
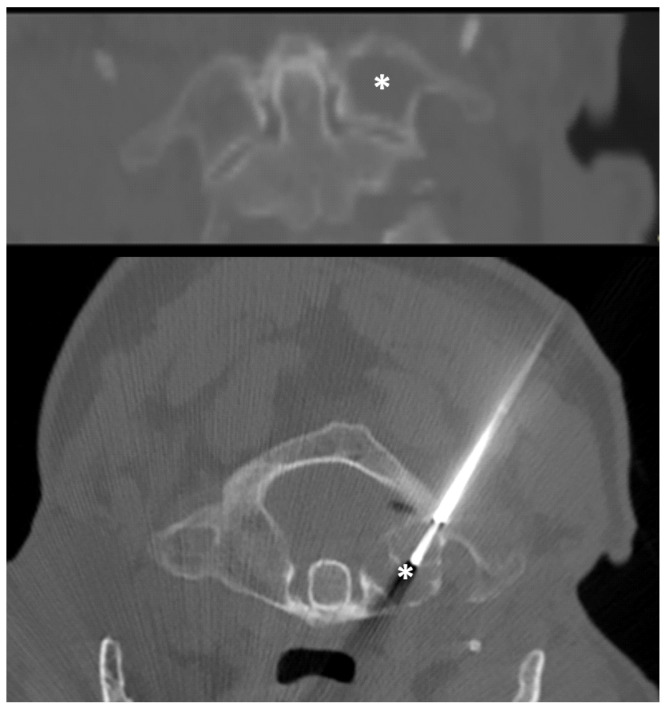
A 73-year-old man. CT frontal view (**Up**) showed a mixed lesion (mostly lytic, shown in asterisks) of the left lateral mass of the atlas. CT-guided biopsy was performed with a posterior approach (**Down**). The procedure resulted in the diagnosis of metastasis of prostate cancer.

All of the procedures were performed with patients in supine or prone position; no anterior/trans-oral approach was performed in this series. In 2 patients (2/16—12.5%), a lateral approach was chosen, while in the remaining 14 patients (87.5%), a posterior approach was carried out (Figure 1 and Figure 2). 

**Figure 3:**
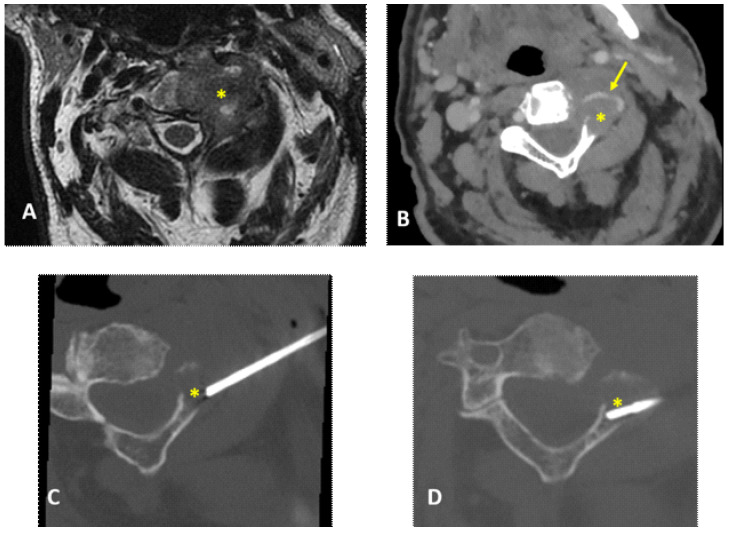
A 79-year-old man: MRI T2w axial (**A**) showed an osteolytic lesion of the axis involving the body and left part of posterior arch (asterisks). Pre-procedural CT scan with intravenous contrast media injection (**B**) was able to define the position of the left vertebral artery (arrow) inside of the target lesion. CT-guided biopsy performed with lateral approach was technically successful with the needle placed within the target lesion (**C**,**D**), and biopsy specimen was obtained. CT-guided biopsy (**C**) was performed with a posterior approach using a 14G needle (Bonopty). The final histopathological diagnosis on biopsy specimen was multiple myeloma.

**Figure 4:**
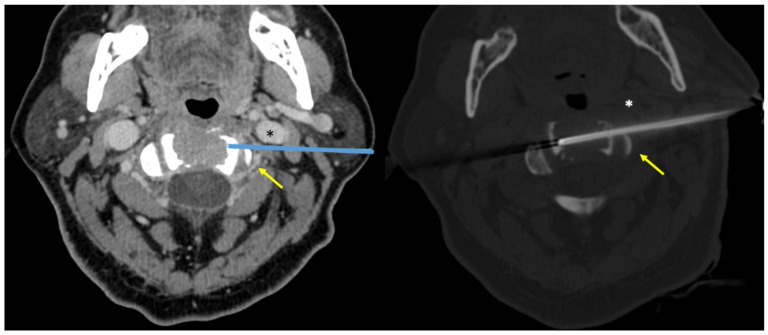
A 64-year-old woman. Preliminary CT scan with intravenous contrast injection, axial view (**Left**), allowed for planning the biopsy approach (**Right**). Vital vascular structures can be visualized: left vertebral artery (arrows) and left jugular vein (asterisks). The biopsy allowed obtaining the histopathological diagnosis as multiple myeloma.

In four patients (4/16—25%), intravenous contrast media injection was necessary before the preliminary CT scan to assess lesion–vascular relationship/proximity; the contrast-enhanced CT scan was always performed in biopsies with lateral needle approach (2/2—100%) (Figure 3 and Figure 4).

The microbiological examinations resulted in negative in all cases (16/16—100%).

The time required for the complete biopsy procedure, including pre-biopsy CT, varied from 30 min to 80 min (median time 45–50 min).

All procedures were performed with local anesthesia and mild sedation. The main patients’ characteristics, biopsy technical data, and histopathological results are summarized in Table 1.

## 4. Discussion

In our series, no procedures were performed using the anterior/trans-oral approach; nevertheless, in the literature, several successful CT-guided biopsies have been reported using this particular approach [12,13,14,15,16]. Moreover, in some specific locations within the CVJ area (e.g., the dens of the axis), the anterior/trans-oral approach could be the only feasible and safe one to the target lesion. This particular approach is affected by the need for general anesthesia [14], and by the more laborious trans-oral mouth open position. This approach is feasible also with fluoroscopic guidance, while in the posterior and lateral ones, CT guidance is necessary [13]. According to our experience and literature data, the posterior or lateral approach to the CVJ area under CT guidance, when feasible, should be preferred to the anterior trans-oral one [13].

In cases in which vertebral arteries position is not clearly recognizable on preliminary unenhanced CT scans, the use of intravenous contrast media injection is mandatory to depict these vessels and perform the biopsy with a safe approach avoiding serious complications. In cases of lateral approach, intravenous contrast media injection is always recommended [13]. Indeed, in our series, both lesions laterally approached (2/2—100%) were preliminary evaluated with contrast-enhanced CT.

A recent interesting systematic review focused on CT-guided spinal biopsies analyzed 39 studies with 3917 total patients [2]. The authors found an overall diagnostic accuracy per procedure of 86% (95% CI 82% to 89%), slightly higher than the one achieved in our series of biopsies of the CVJ area (81%). The complication rate account for only 1% (95% CI 0.4% to 1.9%). Transient post-procedural pain and minor hematoma were the most common complications that occurred [2]. In our series, no complications were observed, even though these data were limited to a small sample. 

The diagnostic accuracy of our series is also lower than the one obtained by Wiesner et al. in a large series of 74 cervical biopsies (81% versus 96%) [9]. Despite the slightly lower diagnostic success of CT-guided biopsy of the CVJ of our series, compared with other studies focused on different locations, we suggest that CT-guided biopsy should be the first approach for histopathological assessment of bone lesions, even in this site. This is justified by the safety of the procedure and the less invasive approach, compared with open surgical biopsy.

Our series revealed that several common skeletal neoplasms may affect the CVJ region. Multiple myeloma and metastases were the two most common diagnoses, both accounting for 25% of cases. This possible uncommon location for these systemic diseases has been already reported in the literature [17,18,19,20]. In our series, microbiological results were negative in all cases; nevertheless, this skeletal site may be also affected by different kinds of infections, particularly tuberculosis [21,22,23]. Due to this fact, collecting at least one sample for microbiological analyses is recommended.

To the best of our knowledge, this is the first study aimed at the assessment of the safety and effectiveness of CT-guided biopsy for bone lesions located within the CVJ area. 

CVJ is a complex and critical area of the skeleton. It has been considered for decades as the “land of no one” since the surgical or percutaneous approaches were extremely limited among physicians of different subspecialties [24]. The precision and anatomical detail obtained with CT studies allow performing image-guided biopsies in a safe manner, even in this critical region. Physicians designated to perform these procedures should be adequately trained, as suggested also by other similar studies focused on the spine [25]. Indeed, the knowledge of the anatomy of this tract and its recognition on radiological images is crucial to plan and perform these peculiar CT-guided biopsies.

### Limitations of the Study

This study presents several limitations. This is a retrospective series of 16 patients only. Indeed, the major limits of the current study are represented by the retrospective nature of the research and by the small samples of subjects included. We did not record how many biopsy requests were considered not safely feasible and, consequently, were not performed. Moreover, we did not have the final histopathological diagnosis of the three patients for whom the biopsy was found to be not diagnostic.

## 5. Conclusions

CT-guided biopsy of bone lesions located into the CVJ area was found to be safe in our series. Moreover, this procedure permitted obtaining the diagnosis in most cases. Due to the minimally invasive approach and short time of hospitalization for these kinds of procedures, CT-guided biopsy has the potential to be the first-line diagnostic tool for assessment of histology in bone lesions even with a location in risky skeletal sites such as the CVJ area.

## Figures and Tables

**Table 1:** Main characteristics of patients, lesions, technical data, and biopsy results. (C0 = occiput C1 = atlas C2 = axis).

Patient n°	Age, Sex	Lesion Location	Radiologic Feature	Needle Gauge	Intravenous Contrast Media Injection	Biopsy Approach	Histological Diagnosis on Biopsy Specimen
1	18, M	C2	Osteolytic	14	No	Posterior	Aneurismal bone cyst
2	71, M	C2	Osteolytic	14	No	Posterior	Multiple myeloma
3	49, M	C1	Osteolytic	14	No	Posterior	Aggressive hemangioma
4	64, F	C2	Osteolytic	14	Yes	Lateral	Multiple myeloma
5	64, F	C1	Osteolytic	14	No	Posterior	Metastasis (breast cancer)
6	40, F	C2	Osteolytic	14	No	Posterior	Metastasis (breast cancer)
7	39, M	C1	Osteolytic	14	No	Posterior	Multiple myeloma
8	16, M	C2	Osteolytic	14	No	Posterior	Aneurismal bone cyst
9	79, M	C2	Osteolytic	14	Yes	Lateral	Non-diagnostic
10	23, M	C1	Osteolytic	14	No	Posterior	Blood and fibrin (non-diagnostic)
11	73, M	C1	Mixed	14	No	Posterior	Metastasis (prostate cancer)
12	52, F	C0 and C1	Osteolytic	14	Yes	Posterior	Multiple myeloma
13	28, F	C1	Osteolytic	14	No	Posterior	Aneurismal bone cyst
14	72, F	C1 and C2	Sclerotic	12	No	Posterior	Pseudogout
15	51, F	C1 and C2	Osteolytic	14	Yes	Posterior	Rare giant cells (non-diagnostic)
16	86, F	C0	Osteolytic	12	No	Posterior	Metastasis (breast cancer)

## Data Availability

The data presented in this study are available on request from the corresponding author.
